# Supplementary material for: Rapid and highly efficient morphogenic gene-mediated hexaploid wheat transformation
Source: Front Plant Sci. 2023 Mar 29;14:1151762. doi: 10.3389/fpls.2023.1151762 (PMC10090459; doi:10.3389/fpls.2023.1151762)
Supplement: Supplementary file 1 [file DataSheet_1.pdf]

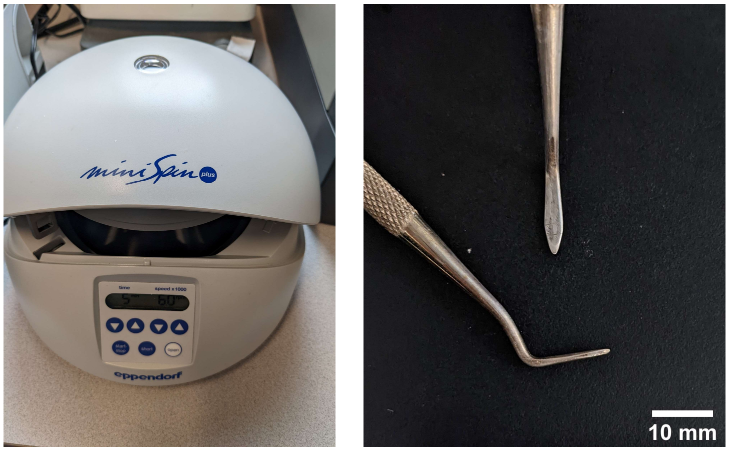

Supplemental Figure 1: Tools used for wheat transformation
(A) Tabletop centrifuge (B) Embryo Isolation Tool

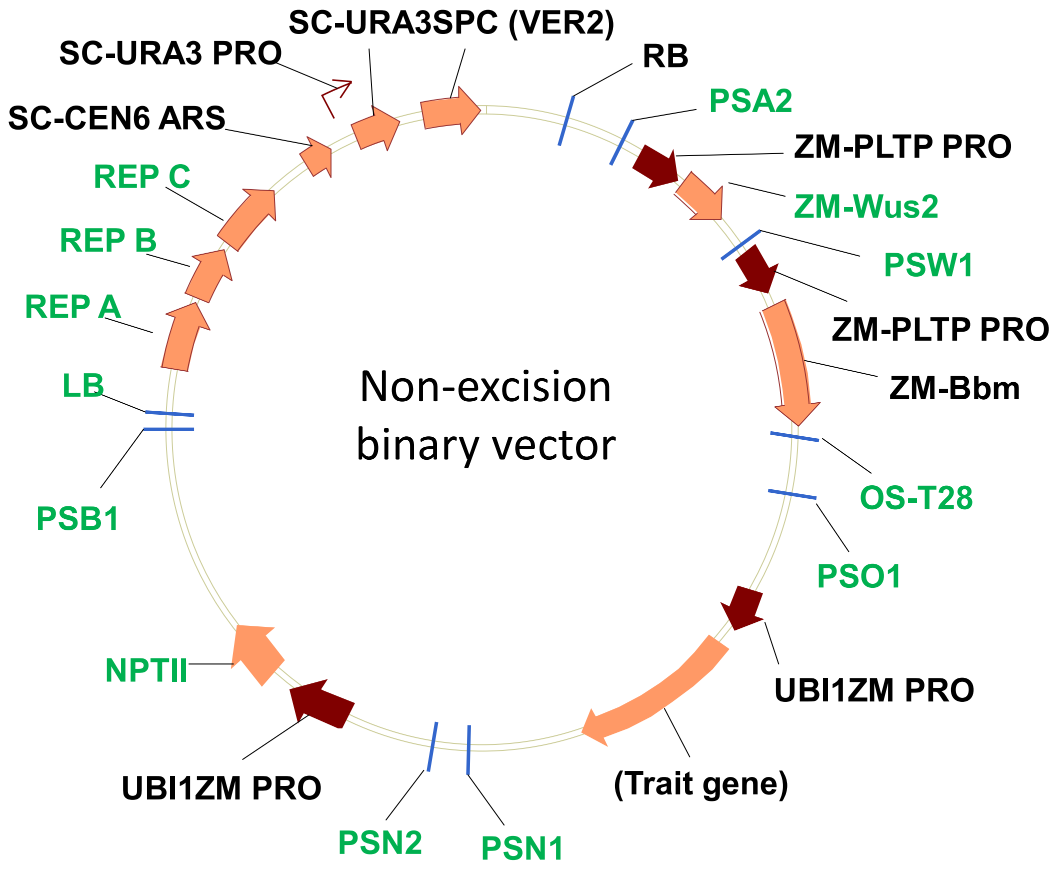

Supplemental Figure 2: Schematic representation of the qPCR assays used in this study.
Event quality and dependency were analyzed by 13 assays (in green) .

Supplementary Table 1: Event Dependency Determination

Embryo	Individual Plantlet	QE Call	Event Dependency Call	PSA2	NPTII	PSB1	ZM-WUS2	PSW1	ZM-BBM	PSO1	PSN1	PSN2	LB	REP A	REP B	REP C
1	1a	Non-QE	Non-Clonal	NULL	1	1	NEGATIVE	NEGATIVE	NEGATIVE	NEGATIVE	NEGATIVE	NEGATIVE	NEGATIVE	NEGATIVE	NEGATIVE	NEGATIVE
	1b	QE	Non-Clonal	1	1	1	POSITIVE	POSITIVE	POSITIVE	POSITIVE	POSITIVE	POSITIVE	NEGATIVE	NEGATIVE	NEGATIVE	NEGATIVE
	1c	Non-QE	Non-Clonal	2	2	3	POSITIVE	POSITIVE	POSITIVE	POSITIVE	POSITIVE	POSITIVE	NEGATIVE	NEGATIVE	NEGATIVE	NEGATIVE
2	2a	Non-QE	Non-Clonal	1	2	3	POSITIVE	POSITIVE	POSITIVE	POSITIVE	POSITIVE	POSITIVE	NEGATIVE	NEGATIVE	NEGATIVE	NEGATIVE
	2b	Non-QE	Non-Clonal	3	1	1	POSITIVE	POSITIVE	POSITIVE	POSITIVE	POSITIVE	POSITIVE	NEGATIVE	NEGATIVE	NEGATIVE	NEGATIVE
	2c	QE	Non-Clonal	1	1	1	POSITIVE	POSITIVE	POSITIVE	POSITIVE	POSITIVE	POSITIVE	NEGATIVE	NEGATIVE	NEGATIVE	NEGATIVE
	2d	Non-QE	Non-Clonal	NULL	1	1	NEGATIVE	NEGATIVE	NEGATIVE	NEGATIVE	POSITIVE	POSITIVE	NEGATIVE	NEGATIVE	NEGATIVE	NEGATIVE
3	3a	Non-QE	Non-Clonal	4	3	1	POSITIVE	POSITIVE	POSITIVE	POSITIVE	POSITIVE	POSITIVE	NEGATIVE	NEGATIVE	NEGATIVE	NEGATIVE
	3b	Non-QE	Non-Clonal	2	2	1	POSITIVE	POSITIVE	POSITIVE	POSITIVE	POSITIVE	POSITIVE	NEGATIVE	NEGATIVE	NEGATIVE	NEGATIVE
	3c	Non-QE	Non-Clonal	2	1	1	POSITIVE	POSITIVE	POSITIVE	POSITIVE	POSITIVE	POSITIVE	NEGATIVE	NEGATIVE	NEGATIVE	NEGATIVE
4	4a	Non-QE	Non-Clonal	1	1	1	POSITIVE	POSITIVE	POSITIVE	POSITIVE	POSITIVE	POSITIVE	POSITIVE	POSITIVE	POSITIVE	POSITIVE
	4b	Non-QE	Non-Clonal	4	3	4	POSITIVE	POSITIVE	POSITIVE	POSITIVE	POSITIVE	POSITIVE	NEGATIVE	NEGATIVE	NEGATIVE	NEGATIVE
	4c	Non-QE	Non-Clonal	1	1	2	POSITIVE	POSITIVE	POSITIVE	POSITIVE	POSITIVE	POSITIVE	NEGATIVE	NEGATIVE	NEGATIVE	NULL
	4d	Non-QE	Non-Clonal	2	1	1	POSITIVE	POSITIVE	POSITIVE	POSITIVE	POSITIVE	POSITIVE	NEGATIVE	NEGATIVE	NEGATIVE	NEGATIVE
	4e	Non-QE	Non-Clonal	4	2	2	POSITIVE	POSITIVE	POSITIVE	POSITIVE	POSITIVE	POSITIVE	NEGATIVE	NEGATIVE	NEGATIVE	NEGATIVE
	4f	QE	Non-Clonal	1	1	1	POSITIVE	POSITIVE	POSITIVE	POSITIVE	POSITIVE	POSITIVE	NEGATIVE	NEGATIVE	NEGATIVE	NEGATIVE
5	5a	Non-QE	Non-Clonal	2	1	1	POSITIVE	POSITIVE	POSITIVE	POSITIVE	POSITIVE	POSITIVE	NEGATIVE	NEGATIVE	NEGATIVE	NEGATIVE
	5b	QE	Non-Clonal	1	1	1	POSITIVE	POSITIVE	POSITIVE	POSITIVE	POSITIVE	POSITIVE	NEGATIVE	NEGATIVE	NEGATIVE	NEGATIVE
6	6a	Non-QE	Non-Clonal	2	1	1	POSITIVE	POSITIVE	POSITIVE	POSITIVE	POSITIVE	POSITIVE	NEGATIVE	NEGATIVE	NEGATIVE	NEGATIVE
	6b	QE	Non-Clonal	1	1	1	POSITIVE	POSITIVE	POSITIVE	POSITIVE	POSITIVE	POSITIVE	NEGATIVE	NEGATIVE	NEGATIVE	NEGATIVE
7	7a	Non-QE	Non-Clonal	2	2	3	POSITIVE	POSITIVE	POSITIVE	POSITIVE	POSITIVE	POSITIVE	NEGATIVE	NEGATIVE	NEGATIVE	NEGATIVE
	7b	Non-QE	Non-Clonal	1	1	3	POSITIVE	POSITIVE	POSITIVE	POSITIVE	POSITIVE	POSITIVE	NEGATIVE	NEGATIVE	NEGATIVE	NEGATIVE
8	8a	Non-QE	Non-Clonal	1	2	2	POSITIVE	POSITIVE	POSITIVE	POSITIVE	POSITIVE	POSITIVE	NEGATIVE	NEGATIVE	NEGATIVE	NEGATIVE
	8b	Non-QE	Non-Clonal	3	2	2	POSITIVE	POSITIVE	POSITIVE	POSITIVE	POSITIVE	POSITIVE	NEGATIVE	NEGATIVE	NEGATIVE	NEGATIVE
9	9a	Non-QE	Non-Clonal	2	2	2	POSITIVE	POSITIVE	POSITIVE	POSITIVE	POSITIVE	POSITIVE	NEGATIVE	NEGATIVE	NEGATIVE	NEGATIVE
	9b	QE	Non-Clonal	1	1	1	POSITIVE	POSITIVE	POSITIVE	POSITIVE	POSITIVE	POSITIVE	NEGATIVE	NEGATIVE	NEGATIVE	NEGATIVE
10	10a	QE	Non-Clonal	1	1	1	POSITIVE	POSITIVE	POSITIVE	POSITIVE	POSITIVE	POSITIVE	NEGATIVE	NEGATIVE	NEGATIVE	NEGATIVE
	10b	Non-QE	Non-Clonal	NULL	1	1	POSITIVE	POSITIVE	POSITIVE	POSITIVE	POSITIVE	POSITIVE	NEGATIVE	NEGATIVE	NEGATIVE	NEGATIVE
11	11a	Non-QE	Non-Clonal	1	1	1	NEGATIVE	NEGATIVE	NEGATIVE	NEGATIVE	POSITIVE	POSITIVE	NEGATIVE	NEGATIVE	NEGATIVE	NEGATIVE
	11b	Non-QE	Non-Clonal	4	2	2	POSITIVE	POSITIVE	POSITIVE	POSITIVE	POSITIVE	POSITIVE	NEGATIVE	NEGATIVE	NEGATIVE	NEGATIVE
12	12a	Non-QE	Non-Clonal	3	2	3	POSITIVE	POSITIVE	POSITIVE	POSITIVE	POSITIVE	POSITIVE	NEGATIVE	NEGATIVE	NEGATIVE	NEGATIVE
	12b	Non-QE	Non-Clonal	2	1	2	POSITIVE	POSITIVE	POSITIVE	POSITIVE	POSITIVE	POSITIVE	NEGATIVE	NEGATIVE	NEGATIVE	NEGATIVE
13	13a	Non-QE	Non-Clonal	2	3	3	POSITIVE	POSITIVE	POSITIVE	POSITIVE	POSITIVE	POSITIVE	NEGATIVE	NEGATIVE	NEGATIVE	NEGATIVE
	13b	Non-QE	Non-Clonal	3	3	2	POSITIVE	POSITIVE	POSITIVE	POSITIVE	POSITIVE	POSITIVE	NEGATIVE	NEGATIVE	NEGATIVE	NEGATIVE
	13c	Non-QE	Non-Clonal	4	2	2	POSITIVE	POSITIVE	POSITIVE	POSITIVE	POSITIVE	POSITIVE	POSITIVE	POSITIVE	POSITIVE	POSITIVE
14	14a	Non-QE	Non-Clonal	1	2	2	POSITIVE	POSITIVE	POSITIVE	POSITIVE	POSITIVE	POSITIVE	POSITIVE	POSITIVE	POSITIVE	POSITIVE
	14b	QE	Non-Clonal	1	1	1	POSITIVE	POSITIVE	POSITIVE	POSITIVE	POSITIVE	POSITIVE	NEGATIVE	NEGATIVE	NEGATIVE	NEGATIVE
15	15a	Non-QE	Non-Clonal	3	3	3	POSITIVE	POSITIVE	POSITIVE	POSITIVE	POSITIVE	POSITIVE	NEGATIVE	NEGATIVE	NEGATIVE	NEGATIVE
	15b	Non-QE	Non-Clonal	3	2	1	POSITIVE	POSITIVE	POSITIVE	POSITIVE	POSITIVE	POSITIVE	NEGATIVE	NEGATIVE	NEGATIVE	NEGATIVE
16	16a	Non-QE	Non-Clonal	3	1	1	POSITIVE	POSITIVE	POSITIVE	POSITIVE	POSITIVE	POSITIVE	NEGATIVE	NEGATIVE	NEGATIVE	NEGATIVE
	16b	QE	Non-Clonal	1	1	1	POSITIVE	POSITIVE	POSITIVE	POSITIVE	POSITIVE	POSITIVE	NEGATIVE	NEGATIVE	NEGATIVE	NEGATIVE
	16c	Non-QE	Non-Clonal	2	1	2	POSITIVE	POSITIVE	POSITIVE	POSITIVE	POSITIVE	POSITIVE	NEGATIVE	NEGATIVE	NEGATIVE	NEGATIVE
17	17a	Non-QE	Non-Clonal	1	1	2	POSITIVE	POSITIVE	POSITIVE	POSITIVE	POSITIVE	POSITIVE	NEGATIVE	NEGATIVE	NEGATIVE	NEGATIVE
	17b	Non-QE	Non-Clonal	1	1	NULL	POSITIVE	POSITIVE	POSITIVE	POSITIVE	POSITIVE	POSITIVE	NEGATIVE	NEGATIVE	NEGATIVE	NEGATIVE
18	18a	Non-QE	Non-Clonal	2	1	1	POSITIVE	POSITIVE	POSITIVE	POSITIVE	POSITIVE	POSITIVE	NEGATIVE	NEGATIVE	NEGATIVE	NEGATIVE
	18b	QE	Non-Clonal	1	1	1	POSITIVE	POSITIVE	POSITIVE	POSITIVE	POSITIVE	POSITIVE	NEGATIVE	NEGATIVE	NEGATIVE	NEGATIVE
19	19a	Non-QE	Non-Clonal	4	2	2	POSITIVE	POSITIVE	POSITIVE	POSITIVE	POSITIVE	POSITIVE	NEGATIVE	NEGATIVE	NEGATIVE	NEGATIVE
	19b	Non-QE	Non-Clonal	2	1	1	POSITIVE	POSITIVE	POSITIVE	POSITIVE	POSITIVE	POSITIVE	NEGATIVE	NEGATIVE	NEGATIVE	NEGATIVE
	19c	QE	Non-Clonal	1	1	1	POSITIVE	POSITIVE	POSITIVE	POSITIVE	POSITIVE	POSITIVE	NEGATIVE	NEGATIVE	NEGATIVE	NEGATIVE
	19d	Non-QE	Non-Clonal	4	3	4	POSITIVE	POSITIVE	POSITIVE	POSITIVE	POSITIVE	POSITIVE	NEGATIVE	NEGATIVE	NEGATIVE	NEGATIVE

Embryo	Individual Plantlet	QE Call	Event Dependency Call	PSA2	NPTII	PSB1	ZM-WUS2	PSW1	ZM-BBM	PSO1	PSN1	PSN2	LB	REP A	REP B	REP C
20	20a	Non-QE	Non-Clonal	2	1	1	POSITIVE	POSITIVE	POSITIVE	POSITIVE	POSITIVE	POSITIVE	NEGATIVE	NEGATIVE	NEGATIVE	NEGATIVE
	20b	Non-QE	Non-Clonal	4	1	1	POSITIVE	POSITIVE	POSITIVE	POSITIVE	POSITIVE	POSITIVE	NEGATIVE	NEGATIVE	NEGATIVE	NEGATIVE
21	21a	Non-QE	Non-Clonal	1	1	2	POSITIVE	POSITIVE	POSITIVE	POSITIVE	POSITIVE	POSITIVE	NEGATIVE	NEGATIVE	NEGATIVE	NEGATIVE
	21b	Non-QE	Non-Clonal	>4COPY	4	4	POSITIVE	POSITIVE	POSITIVE	POSITIVE	POSITIVE	POSITIVE	POSITIVE	POSITIVE	POSITIVE	POSITIVE
22	22a	Non-QE	Non-Clonal	4	3	>4COPY	POSITIVE	POSITIVE	POSITIVE	POSITIVE	POSITIVE	POSITIVE	NEGATIVE	NEGATIVE	NEGATIVE	NEGATIVE
	22b	Non-QE	Non-Clonal	1	1	2	POSITIVE	POSITIVE	POSITIVE	POSITIVE	POSITIVE	POSITIVE	NEGATIVE	NEGATIVE	NEGATIVE	NEGATIVE
	22c	Non-QE	Non-Clonal	4	2	2	POSITIVE	POSITIVE	POSITIVE	POSITIVE	POSITIVE	POSITIVE	NEGATIVE	NEGATIVE	NEGATIVE	NEGATIVE
23	23a	Non-QE	Non-Clonal	2	2	2	POSITIVE	POSITIVE	POSITIVE	POSITIVE	POSITIVE	POSITIVE	NEGATIVE	NEGATIVE	NEGATIVE	NEGATIVE
	23b	QE	Non-Clonal	1	1	1	POSITIVE	POSITIVE	POSITIVE	POSITIVE	POSITIVE	POSITIVE	NEGATIVE	NEGATIVE	NEGATIVE	NEGATIVE
24	24a	QE	Non-Clonal	1	1	1	POSITIVE	POSITIVE	POSITIVE	POSITIVE	POSITIVE	POSITIVE	NEGATIVE	NEGATIVE	NEGATIVE	NEGATIVE
	24b	Non-QE	Non-Clonal	2	1	3	POSITIVE	POSITIVE	POSITIVE	POSITIVE	POSITIVE	POSITIVE	NEGATIVE	NEGATIVE	NEGATIVE	NEGATIVE
	24c	Non-QE	Non-Clonal	4	2	2	POSITIVE	POSITIVE	POSITIVE	POSITIVE	POSITIVE	POSITIVE	NEGATIVE	NEGATIVE	NEGATIVE	NEGATIVE
25	25a	Non-QE	Non-Clonal	2	1	1	POSITIVE	POSITIVE	POSITIVE	POSITIVE	POSITIVE	POSITIVE	NEGATIVE	NEGATIVE	NEGATIVE	NEGATIVE
	25b	Non-QE	Non-Clonal	4	2	1	POSITIVE	POSITIVE	POSITIVE	POSITIVE	POSITIVE	POSITIVE	POSITIVE	NEGATIVE	NEGATIVE	NEGATIVE
	25c	Non-QE	Non-Clonal	1	2	3	POSITIVE	POSITIVE	POSITIVE	POSITIVE	POSITIVE	POSITIVE	NEGATIVE	NEGATIVE	NEGATIVE	NEGATIVE
26	26a	Non-QE	Non-Clonal	4	2	1	POSITIVE	POSITIVE	POSITIVE	POSITIVE	POSITIVE	POSITIVE	NEGATIVE	POSITIVE	NEGATIVE	NEGATIVE
	26b	Non-QE	Non-Clonal	2	1	NULL	POSITIVE	POSITIVE	POSITIVE	POSITIVE	POSITIVE	POSITIVE	NEGATIVE	NEGATIVE	NEGATIVE	NEGATIVE
27	27a	Non-QE	Non-Clonal	1	1	NULL	POSITIVE	POSITIVE	POSITIVE	POSITIVE	POSITIVE	POSITIVE	NEGATIVE	NEGATIVE	NEGATIVE	NEGATIVE
	27b	Non-QE	Non-Clonal	2	1	1	POSITIVE	POSITIVE	POSITIVE	POSITIVE	POSITIVE	POSITIVE	POSITIVE	POSITIVE	POSITIVE	POSITIVE
28	28a	Non-QE	Non-Clonal	2	1	NULL	POSITIVE	POSITIVE	POSITIVE	POSITIVE	POSITIVE	POSITIVE	NEGATIVE	NEGATIVE	NEGATIVE	NEGATIVE
	28b	Non-QE	Non-Clonal	2	1	1	POSITIVE	POSITIVE	POSITIVE	POSITIVE	POSITIVE	POSITIVE	NEGATIVE	NEGATIVE	NEGATIVE	NEGATIVE
	28c	Non-QE	Non-Clonal	3	3	2	POSITIVE	POSITIVE	POSITIVE	POSITIVE	POSITIVE	POSITIVE	NEGATIVE	NEGATIVE	NEGATIVE	NEGATIVE
	28d	Non-QE	Non-Clonal	1	1	NULL	POSITIVE	POSITIVE	POSITIVE	POSITIVE	POSITIVE	POSITIVE	NEGATIVE	NEGATIVE	NEGATIVE	NEGATIVE
29	29a	Non-QE	Presumably Clonal	4	4	4	POSITIVE	POSITIVE	POSITIVE	POSITIVE	POSITIVE	POSITIVE	NEGATIVE	NEGATIVE	NEGATIVE	NEGATIVE
	29b	Non-QE		4	4	4	POSITIVE	POSITIVE	POSITIVE	POSITIVE	POSITIVE	POSITIVE	NEGATIVE	NEGATIVE	NEGATIVE	NEGATIVE
	29c	Non-QE	Non-Clonal	1	1	2	POSITIVE	POSITIVE	POSITIVE	POSITIVE	POSITIVE	POSITIVE	NEGATIVE	NEGATIVE	NEGATIVE	NEGATIVE
30	30a	Non-QE	Presumably Clonal	3	2	2	POSITIVE	POSITIVE	POSITIVE	POSITIVE	POSITIVE	POSITIVE	NEGATIVE	NEGATIVE	NEGATIVE	NEGATIVE
	30b	Non-QE		3	2	2	POSITIVE	POSITIVE	POSITIVE	POSITIVE	POSITIVE	POSITIVE	NEGATIVE	NEGATIVE	NEGATIVE	NEGATIVE
	30c	QE	Presumably Clonal	1	1	1	POSITIVE	POSITIVE	POSITIVE	POSITIVE	POSITIVE	POSITIVE	NEGATIVE	NEGATIVE	NEGATIVE	NEGATIVE
	30d	QE		1	1	1	POSITIVE	POSITIVE	POSITIVE	POSITIVE	POSITIVE	POSITIVE	NEGATIVE	NEGATIVE	NEGATIVE	NEGATIVE
	30e	Non-QE	Non-Clonal	3	2	1	POSITIVE	POSITIVE	POSITIVE	POSITIVE	POSITIVE	POSITIVE	NEGATIVE	NEGATIVE	NEGATIVE	NEGATIVE
	30f	Non-QE	Non-Clonal	2	1	1	POSITIVE	POSITIVE	POSITIVE	POSITIVE	POSITIVE	POSITIVE	NEGATIVE	NEGATIVE	NEGATIVE	NEGATIVE
	30g	Non-QE	Non-Clonal	3	1	1	POSITIVE	POSITIVE	POSITIVE	POSITIVE	POSITIVE	POSITIVE	NEGATIVE	NEGATIVE	NEGATIVE	NEGATIVE
31	31a	Non-QE	Presumably Clonal	2	1	1	POSITIVE	POSITIVE	POSITIVE	POSITIVE	POSITIVE	POSITIVE	NEGATIVE	NEGATIVE	NEGATIVE	NEGATIVE
	31b	Non-QE		2	1	1	POSITIVE	POSITIVE	POSITIVE	POSITIVE	POSITIVE	POSITIVE	NEGATIVE	NEGATIVE	NEGATIVE	NEGATIVE
	31c	Non-QE	Non-Clonal	2	1	2	POSITIVE	POSITIVE	POSITIVE	POSITIVE	POSITIVE	POSITIVE	NEGATIVE	NEGATIVE	NEGATIVE	NEGATIVE
	31d	Non-QE	Non-Clonal	1	1	WEAK POSITIVE	POSITIVE	POSITIVE	POSITIVE	POSITIVE	POSITIVE	POSITIVE	NEGATIVE	NEGATIVE	NEGATIVE	NEGATIVE
	31e	Non-QE	Non-Clonal	>4COPY	>4COPY	3	POSITIVE	POSITIVE	POSITIVE	POSITIVE	POSITIVE	POSITIVE	POSITIVE	POSITIVE	POSITIVE	POSITIVE
32	32a	Non-QE	Presumably Clonal	1	1	2	POSITIVE	POSITIVE	POSITIVE	POSITIVE	POSITIVE	POSITIVE	NEGATIVE	NEGATIVE	NEGATIVE	NEGATIVE
	32b	Non-QE		1	1	2	POSITIVE	POSITIVE	POSITIVE	POSITIVE	POSITIVE	POSITIVE	NEGATIVE	NEGATIVE	NEGATIVE	NEGATIVE
	32c	QE	Non-Clonal	1	1	1	POSITIVE	POSITIVE	POSITIVE	POSITIVE	POSITIVE	POSITIVE	NEGATIVE	NEGATIVE	NEGATIVE	NEGATIVE
	32d	Non-QE	Non-Clonal	1	1	3	POSITIVE	POSITIVE	NEGATIVE	NEGATIVE	POSITIVE	POSITIVE	NEGATIVE	NEGATIVE	NEGATIVE	NEGATIVE
33	33a	Non-QE	Presumably Clonal	2	1	1	POSITIVE	POSITIVE	POSITIVE	POSITIVE	POSITIVE	POSITIVE	NEGATIVE	NEGATIVE	NEGATIVE	NEGATIVE
	33b	Non-QE		2	1	1	POSITIVE	POSITIVE	POSITIVE	POSITIVE	POSITIVE	POSITIVE	NEGATIVE	NEGATIVE	NEGATIVE	NEGATIVE
34	34a	Non-QE	Presumably Clonal	2	1	1	POSITIVE	POSITIVE	POSITIVE	POSITIVE	POSITIVE	POSITIVE	NEGATIVE	NEGATIVE	NEGATIVE	NEGATIVE
	34b	Non-QE		2	1	1	POSITIVE	POSITIVE	POSITIVE	POSITIVE	POSITIVE	POSITIVE	NEGATIVE	NEGATIVE	NEGATIVE	NEGATIVE
	34c	Non-QE	Non-Clonal	4	1	NULL	POSITIVE	POSITIVE	POSITIVE	POSITIVE	POSITIVE	POSITIVE	NEGATIVE	NEGATIVE	NEGATIVE	NEGATIVE
35	35a	Non-QE	Presumably Clonal	1	1	NULL	POSITIVE	POSITIVE	POSITIVE	POSITIVE	POSITIVE	POSITIVE	NEGATIVE	NEGATIVE	NEGATIVE	NEGATIVE
	35b	Non-QE		1	1	NULL	POSITIVE	POSITIVE	POSITIVE	POSITIVE	POSITIVE	POSITIVE	NEGATIVE	NEGATIVE	NEGATIVE	NEGATIVE
	35c	Non-QE	Non-Clonal	1	1	NULL	POSITIVE	POSITIVE	POSITIVE	POSITIVE	POSITIVE	POSITIVE	NEGATIVE	NEGATIVE	NEGATIVE	NEGATIVE
